# PERCEPTIONS AND EXPERIENCES OF FAMILY CAREGIVERS OF OLDER FIRST-GENERATION CHINESE AMERICANS WITH DEMENTIA

**DOI:** 10.1093/geroni/igad104.2839

**Published:** 2023-12-21

**Authors:** Erh-Chi Hsu, Jenny Hsin-Chun Tsai, Clarence Spigner, Kannie Chim

**Affiliations:** Johns Hopkins University, Baltimore, Maryland, United States; University of Washington, Seattle, Washington, United States; University of Washington, Seattle, Washington, United States; International Community Health Services, Seattle, Washington, United States

## Abstract

Family caregivers of individuals with dementia are precious assets whose health is related to the health of their care recipients. First-generation Chinese American older adults are a growing population; the research about their family caregivers’ experience is scarce. Guided by the Health Belief Model (HBM) and the National Institute on Minority Health and Health Disparities (NIMHD) framework, this study examined how the Chinese sociocultural background shapes family caregivers’ perception of dementia, caring behaviors, and caring experiences during the COVID-19 pandemic. Data were collected from 16 family caregivers through one-on-one interviews in three languages with the thematic analysis applied. Findings indicated most caregivers could not differentiate dementia from natural cognitive degeneration. Despite the unclarity, the cultural value of filial respect essentially obligated caregivers to unconditionally devote their time and efforts to provide care. Individual coping skills toward care burden were associated with their knowledge, attitude, access to supportive families/friends and reliable community-based organizations (CBOs), and access to government social services. The care recipients’ motivation and functional status affected the difficulty of providing care. The pandemic disturbed families’ care plans and limited community activities. In addition, the heightened Asian xenophobia atmosphere raised concerns for caregivers to be outdoors. Implications for practice include providing culturally appropriate educational resources on dementia care and incorporating CBOs to promote governmental service referral. Larger studies for Chinese American older adults born outside the US and studies of dementia care burden on their family caregivers’ health are needed to address social and health inequities for historically marginalized cohorts.

